# Fe_**3**_O_**4**_/ZIF-8-90 Nanocomposite as a Strategy for Oncological Treatment

**DOI:** 10.1021/acsomega.5c02819

**Published:** 2025-07-03

**Authors:** Julia Fernanda da Costa Araujo, Giovanna Nogueira da Silva Avelino Oliveira Rocha, José Yago Rodrigues Silva, João Victor Ribeiro Rocha, Andris Figueiroa Bakuzis, Severino Alves Junior

**Affiliations:** † Department of Fundamental Chemistry, 28116 Federal University of Pernambuco, Recife, PE 50670-901, Brazil; ‡ Physics Institute, Federal University of Goias, Goiania 74690-900, Brazil

## Abstract

Cancer is one of the leading causes of mortality worldwide, and traditional treatments, such as systemic chemotherapy, often have side effects due to their lack of specificity. This limitation has driven the search for new, more selective, and effective therapeutic strategies. In this context, this study proposes the development of a magnetic nanocarrier with superparamagnetic iron oxide nanoparticles (SPIONs) associated with the metal–organic framework ZIF-8-90, forming the Fe_3_O_4_/ZIF-8-90 nanosystem. The synthesized nanocarrier showed a uniform size distribution, with an average diameter of 97 nm, and could adsorb approximately 13% of the 5-FU load. Fe_3_O_4_/ZIF-8-90 exhibited significant biocompatibility for healthy cells (Vero strain), maintaining 85% cell viability at concentrations of up to 100 μg/mL. In contrast, it showed selective cytotoxicity against breast (MDA-MB-231) and lung (H292) tumor cells. As for its therapeutic potential through application in magnetic hyperthermia, the nanomaterial raised the temperature by up to 5.2 °C in just 10 min, using field amplitude and frequency below the biologically safe limits. In addition, relaxometric characterization also pointed to Fe_3_O_4_/ZIF-8-90 as a promising contrast agent for MRI in T_2_ mode, achieving an *r*_2_ relaxivity of 180.15 mM^–1^ s^–1^. These results consolidate the synthesized Fe_3_O_4_/ZIF-8-90 nanocarrier as a promising theranostic platform, combining therapeutic efficacy and diagnostic functionality for oncological applications.

## Graphical abstract

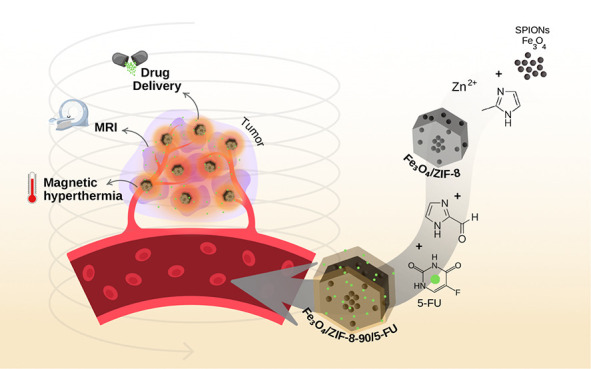

## 1 Introduction

Cancer remains one of the leading causes of death worldwide, posing a significant challenge to public health. According to the Global Cancer Observatory (Globocan), it is estimated that one in five individuals will develop cancer during their lifetime.^1,2^ Despite advancements in conventional treatments such as surgery, radiotherapy, and chemotherapy, these methods often lack specificity, causing collateral damage to healthy tissues and severe adverse effects.^3^ In this context, the search for more effective and less invasive therapeutic approaches has become a priority in the fight against the disease.

In this context, nanotechnology-based systems have emerged as powerful tools for targeted drug delivery, with particular emphasis on metal–organic frameworks (MOFs). MOFs, due to their high porosity, large surface area, and versatile chemistry, have shown exceptional promise in biomedical applications.^4–7^ Among them, Zeolitic Imidazolate Framework-8 (ZIF-8), composed of Zn^2+^ ions and 2-methylimidazole linkers, stands out for its chemical and thermal stability, biocompatibility, high surface area (∼1600 m^2^/g), and tunable pore size (∼11.6 Å), enabling the encapsulation of various therapeutic agents and controlled release in mildly acidic tumor microenvironments.^8–10^

Considering the properties of ZIF-8, structural variants have been investigated to optimize its performance. Among them, ZIF-8-90 stands out, a mixed ligand structure that incorporates 2-imidazolecarboxaldehyde, giving the material additional advantageous characteristics, such as greater hydrophilicity, greater density of surface defects that facilitate interaction with drugs, and greater loading efficiency due to its more polar surface.^11,12^

Studies corroborate the superior performance of ZIF-8-90 compared to ZIF-8. For example, Ma et al. demonstrated that fibers coated with ZIF-8-90 presented high adsorption efficiency for both hydrophilic and hydrophobic targets, an effect attributed to the synergy between the methyl and aldehyde groups on the surface of the material, combined with its high porosity.^11^ Additionally, ZIF-8-90 exhibits greater colloidal stability in aqueous media and better compatibility with water-soluble drugs. In the Yen et al. study, ZIF-90 and its postfunctionalized derivatives showed excellent dispersibility in aqueous solution and significantly lower cytotoxicity when compared to more hydrophobic systems.^13^ Another relevant aspect is the presence of the aldehyde group in its ligand, which acts as an active site for postsynthetic modifications, allowing the conjugation of targeting molecules and expanding its potential for selective delivery into tumor tissues.^14,15^ Thus, ZIF-8-90 represents an evolution over ZIF-8, especially for biological applications.

To introduce additional functionalities and therapeutic synergies, superparamagnetic iron oxide nanoparticles (SPIONs), typically Fe_3_O_4_, have been integrated into metal–organic frameworks (MOFs).^16–19^ SPIONs exhibit excellent biocompatibility,^20–22^ are FDA (Food and Drug Administration) approved for clinical use, and perform multiple functions: as contrast agents in magnetic resonance imaging (MRI),^23,24^ heat mediators in magnetic hyperthermia,^16,25–28^ magnetic drug delivery vehicles,^29^ and also for the treatment of iron deficiency anemia.^30^ Critically, findings have revealed that SPIONs can modulate the tumor immune microenvironment. Zanganeh et al. demonstrated that SPIONs can polarize tumor-associated macrophages (TAMs) toward the pro-inflammatory M1 phenotype, thus activating cytotoxic immune responses and inhibiting tumor growth and metastasis.^31^ Recently, Liu et al. developed an iron-based nanocomposite derived from *Polyporus umbellatus* polysaccharides. This material promoted M1 polarization of TAMs and significantly enhanced antitumor efficacy in a breast cancer model, further confirming the immunomodulatory potential of iron-based systems.^32^ These results highlight the immunomodulatory capacity of SPIONs, positioning them as active agents in cancer therapy.

Beyond their immunomodulatory role, thermal nanomedicine applications of SPIONs also have important oncology applications. When quasi-static superparamagnetic iron oxide NPs are excited by an AC magnetic field, dynamic hysteresis can appear, resulting in local heat release.^33^ This property has led to the clinical approval of magnetic hyperthermia combined with radiotherapy for brain cancer therapy.^33,34^ Heat can also trigger an immune response,^35^ as demonstrated by recent studies,^36–38^ and enhancement effects are expected due to the biodegradation of the NPs, since they release metallic ions that might be relevant for cancer immunotherapy.^37,39^

In this sense, the integration of Fe_3_O_4_ nanoparticles into ZIF-8-90 matrices creates a hybrid nanocomposite that takes advantage of the synergistic benefits of both components: the structural versatility and high load capacity of ZIF-8-90 and the magnetic responsiveness, imaging capability, and immunomodulatory potential of SPIONs. Compared to Fe_3_O_4_/ZIF-8 systems, Fe_3_O_4_/ZIF-8-90 offers improved drug–carrier interactions, higher dispersion stability in biological environments, and therapeutic performance.

In the context of chemotherapy, 5-Fluorouracil (5-FU) is a widely used antimetabolite agent due to its effectiveness against various types of cancer.^40,41^ However, its clinical application faces significant limitations, such as lack of specificity, adverse effects, and the development of drug resistance by tumor cells.^42–44^ Strategies to overcome these limitations, such as the encapsulation of 5-FU in advanced drug delivery systems, can potentially improve its therapeutic efficacy and reduce its side effects significantly.

Therefore, this study aims to synthesize and characterize the Fe_3_O_4_/ZIF-8-90 nanocomposite, as well as its application in the controlled loading and release of the anticancer drug 5-FU. Additionally, the system’s functionalities for magnetic hyperthermia and its potential as a contrast agent in magnetic resonance imaging will be explored, consolidating an integrated and efficient approach to cancer treatment.

## 2 Results and Discussion

### 2.1 Characterizations

The X-ray diffraction (XRD) patterns presented in Figure 1a show the characteristic peaks of the synthesized ZIFs, in addition to the calculated pattern for ZIF-8 (COD 4118891, Crystallography Open Database). The diffractograms confirm the isoreticularity between the ZIF-8 and ZIF-8-90 structures, both based on the coordination of zinc with imidazole-class ligands.^12,45^ This behavior was expected since, as reported in the literature, the structure of the hybrid ZIF-8-90 exhibits structural variations of less than 3% compared to its ZIF-8 and ZIF-90 counterparts.^9^

**Figure 1:**
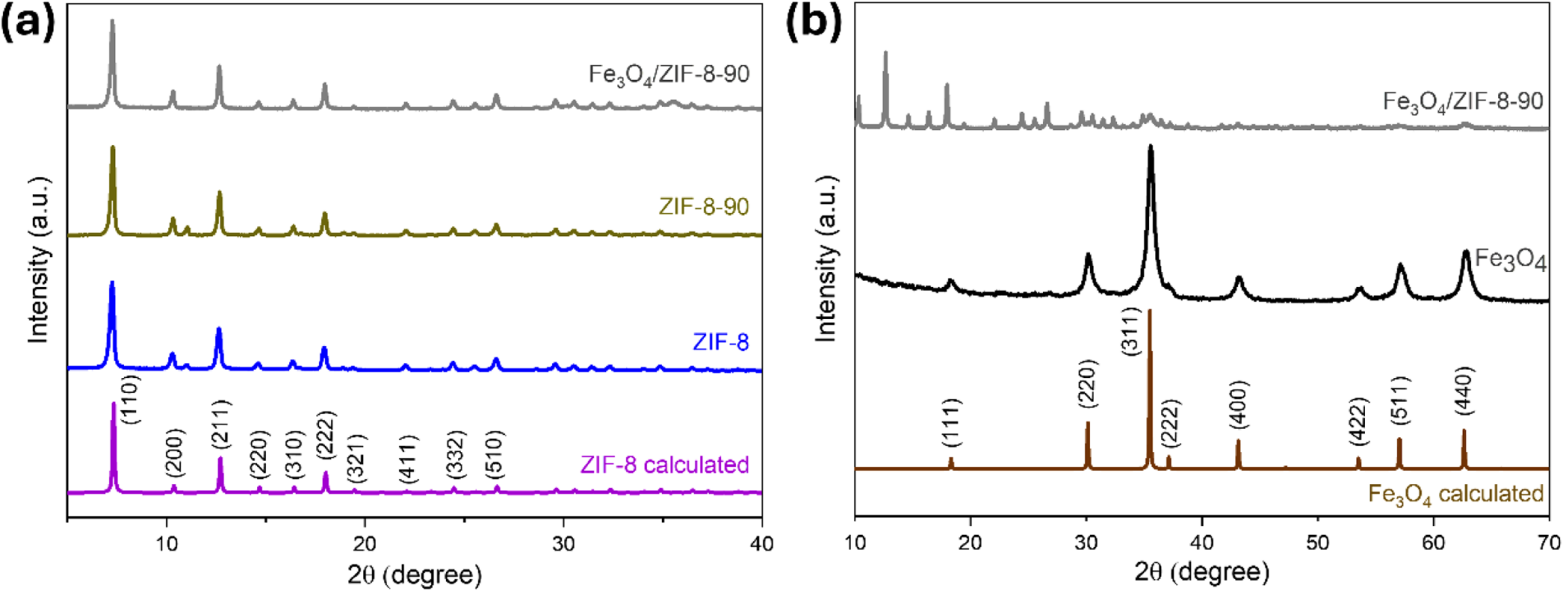
(a) and (b) XRD spectra of prepared ZIF-8, ZIF-8-90, Fe_3_O_4_ and Fe_3_O_4_/ZIF-8-90.

In the case of SPIONs (Figure 1b), the observed diffraction peaks show excellent agreement with the calculated pattern for the magnetite phase (COD 1010369). These peaks are by the works of Siregar et al. and Dutta et al., who observed similar diffractograms with 2θ values around 30.12°, 35.48, 43.12, 53.5, 57.04, and 62.64 corresponding to (220), (311), (400), (422), (511), and (440) planes, respectively.^22,46^ Additionally, in the diffractograms of the Fe_3_O_4_/ZIF-8-90 nanosystem, it is possible to identify the (311) and (400) planes of the SPIONs. These results confirm the successful incorporation of SPIONs into the ZIF-8-90 matrix, consolidating the presence of iron oxide in the hybrid structure.

The refinement of the X-ray diffraction patterns was performed using the GSAS-II software. The collected data calculated crystallographic parameters, and the details of the XRD data refinements are presented in Table S1, and the graphs generated after refinement are in Figure S1.

The phase fraction ratio (Fe_3_O_4_: 41.165%; ZIF-8-90:58.835%) is surprising, as a higher percentage for the hybrid ZIF was expected due to the low intensity of characteristic peaks corresponding to the iron oxide planes in the final material. The SPIONs were identified as an FCC system with space group *Fd*-3m, typical of magnetite, indicating high structural symmetry,^47,48^ and the materials demonstrated its system as BCC and space group *I-*43m.^49^ The unit cell volume, which is 4950.42 Å^3^, is significantly more extensive than that of SPIONs due to large internal cavities in the metal–organic material.

To compare the crystallite size obtained through GSAS-II, the Williamson-Hall and Scherrer methods were also employed.^50–52^ The crystallite size (Table S1) increases with the incorporation of NPs and the formation of the hybrid ZIF (ZIF-8:68.2 nm < ZIF-8-90:70.02 nm < Fe_3_O_4_/ZIF-8-90:106.2 nm), which aligns with expectations and closely matches the values obtained from crystal counting in the SEM images.

In the FTIR spectrum (Figure 2a), characteristic bands were observed, confirming the formation of the metal–organic frameworks ZIF-8 and ZIF-8-90. The band at 420 cm^–1^ is associated with the stretching vibration of the Zn–N bond, confirming the coordination between the imidazolate ligand and the zinc metal ion.^42,53^ In the ZIF-8-90 hybrid, notable bands at 1681 and 790 cm^–1^ were observed, corresponding to the C=O stretching and C–H bending vibrations, respectively, both attributed to the aldehyde group of the ICA ligand, reinforcing the formation of the ZIF-90 structure.^54–57^

**Figure 2:**
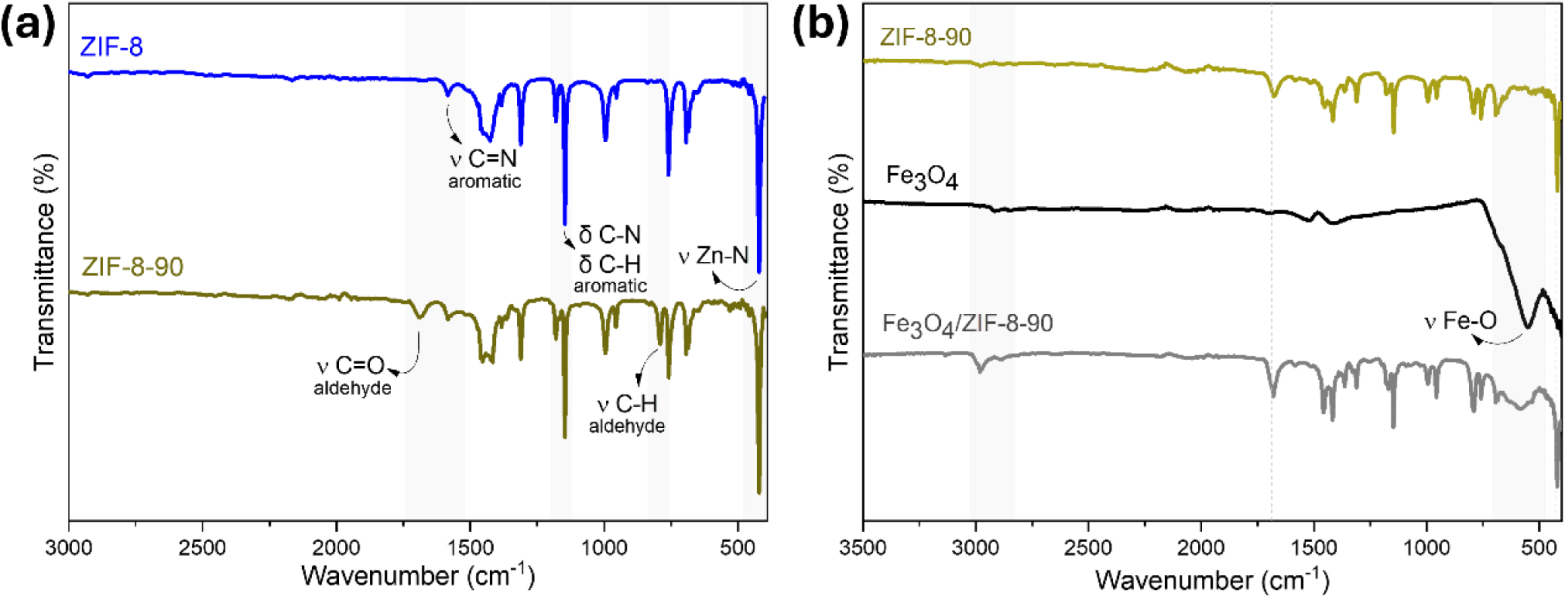
(a) and (b) FT-IR spectra of ZIF-8, ZIF-8-90, Fe_3_O_4_ and Fe_3_O_4_/ZIF-8-90.

In the Fe_3_O_4_ spectrum (Figure 2b), a band at 550 cm^–1^ was identified, attributed to the Fe–O stretching vibrations. This finding aligns with the studies by Beigi and Babamoradi and Kutluay et al., characterizing the spinel structure of magnetite and confirming the presence of iron oxide in the nanocomposite composition.^21,58–60^

The morphologies and structures of Fe_3_O_4_ nanoparticles and the Fe_3_O_4_/ZIF-8-90 nanocomposite are presented in the SEM micrographs in Figure 3. The Fe_3_O_4_ nanoparticles appeared aggregated, with tiny crystals approximately 11 nm in size (Figure 3a). The Fe_3_O_4_/ZIF-8-90 nanocomposite retained its morphology compared to pure ZIF-8-90 (Figure S2b), exhibiting a well-defined orthorhombic structure with smooth and homogeneous surfaces and an average diameter of 97 nm (Figure 3b), a value considered ideal for biological applications in tumor tissues according to Wang et al.^61^ A slight coexistence of polyhedral particles of varying sizes was observed, likely resulting from heterogeneous crystal nucleations on the surfaces of preexisting crystals, a phenomenon known as Ostwald ripening.^62^

**Figure 3:**
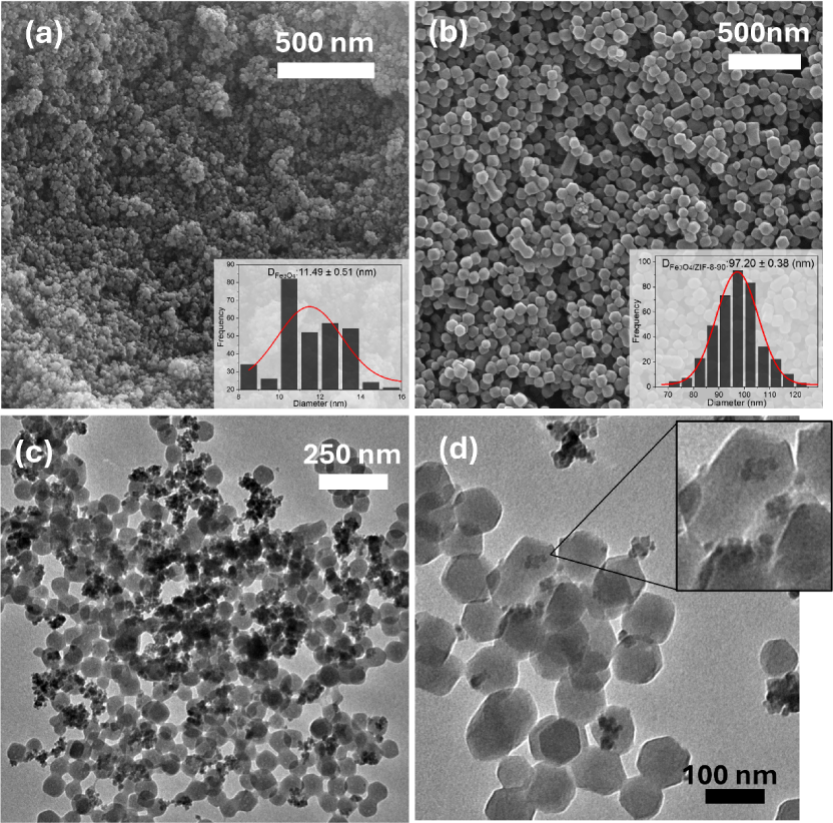
Micrographs of (a) Fe_3_O_4_ and (b) Fe_3_O_4_/ZIF-8-90 obtained by scanning electron microscopy (SEM). Images (c) and (d) correspond to transmission electron microscopy (TEM) of the Fe_3_O_4_ZIF-8-90 system, evidencing the presence of Fe_3_O_4_ nanoparticles inside and on the surface of the ZIF-8-90 crystals.

Transmission electron microscopy (TEM) analysis in Figure 3c revealed that the SPIONs within the sample tended to organize into small clusters, both inside and on the surface of the ZIF-8-90 crystals. This behavior can be attributed to the small size of the Fe_3_O_4_ nanoparticles and the specific interactions between functional groups on the PVP functionalized SPIONs and the ligands of ZIF-8-90, as observed by Lu et al.^63^ In some crystals, SPIONs were centralized (Figure 3d), potentially indicating preferential heterogeneous nucleation during the initial formation of ZIF-8-90 crystals. This result is consistent with studies by Abdelmigeed et al., who reported SPION localization at the core of ZIF-8 crystals, and Chen et al., who observed similar behavior in ZIF-90 crystals, highlighting the impact of these hybrid structures on thermal stability and magnetic properties.^64,65^ TEM analysis of SPIONs are provided in Figure S3.

EDS analysis confirmed the homogeneous distribution of iron throughout the sample, with a relative percentage of 17.2%, complementing the observations made earlier (Table S2). While TEM revealed that SPIONs are not always encapsulated within the ZIF-8-90 crystals, EDS elemental mapping (Figure 4) demonstrated that Fe_3_O_4_ nanoparticles are generally well-distributed across the entire material. Additionally, the presence of characteristic ZIF-8-90 elements such as zinc and nitrogen further support the successful formation of the hybrid system. Additional EDS analyses are included in the appendix (Figure S4).

**Figure 4:**
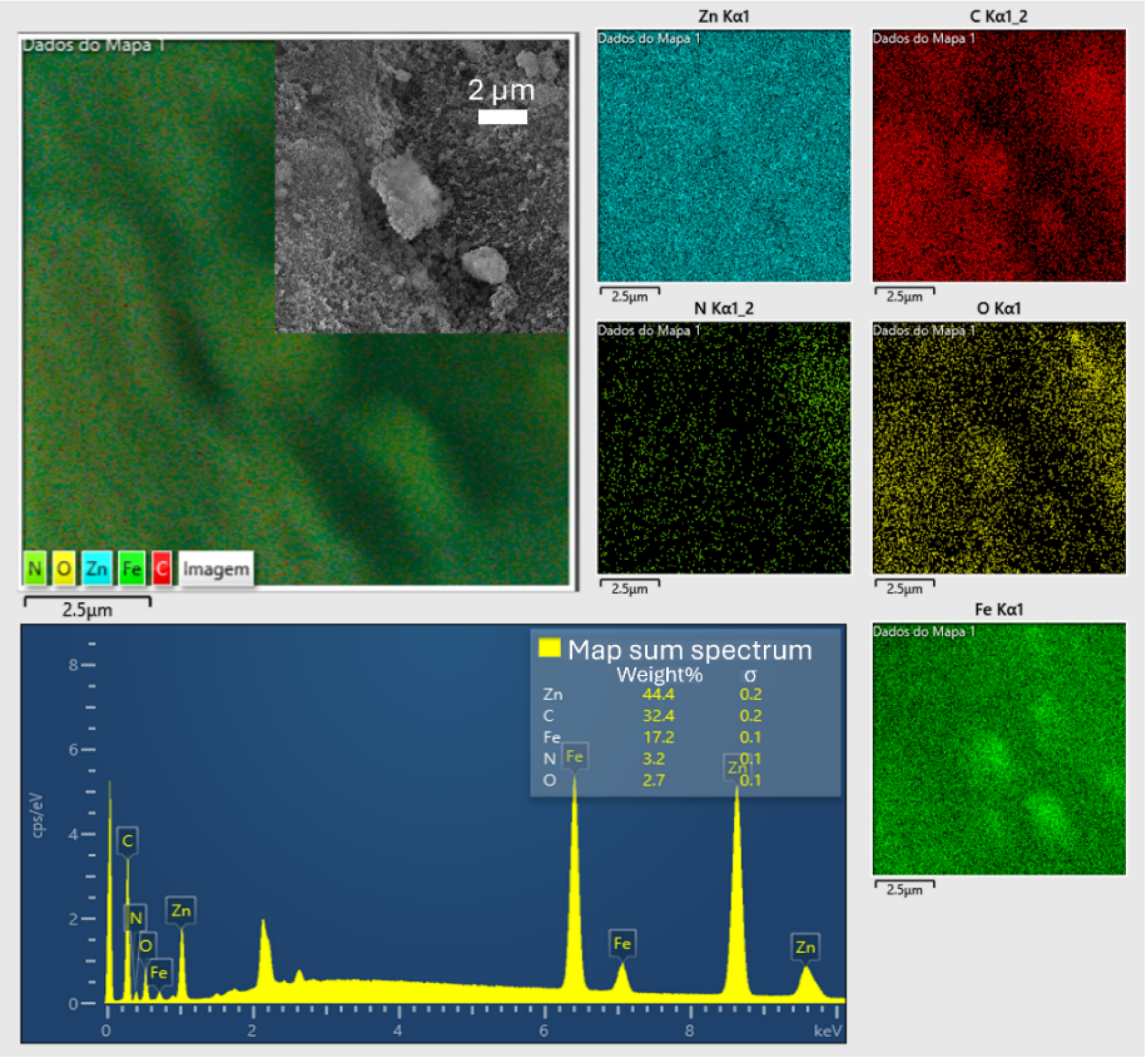
EDS analysis and elemental mapping of the Fe_3_O_4_/ZIF-8-90 nanocomposite.

The thermogravimetric analysis (TGA) of the materials described in this study (Figure 5) was employed to assess their thermal stability and identify the system’s decomposition stages. For the Fe_3_O_4_ nanoparticles stabilized with oleic acid and PVP, a single significant mass loss of 7% was observed starting at 170 °C, attributed to the decomposition of the stabilizing agents. The material demonstrated thermal stability above 400 °C, with an exothermic event at 515 °C (Figure S5c) associated with oxygen reduction in the crystalline lattice. This process, as described by Periakaruppan et al. and Moacă et al., results in the formation of secondary phases such as γ-Fe_2_O_3_ and/or α-Fe_2_O_3_.^66,67^

**Figure 5:**
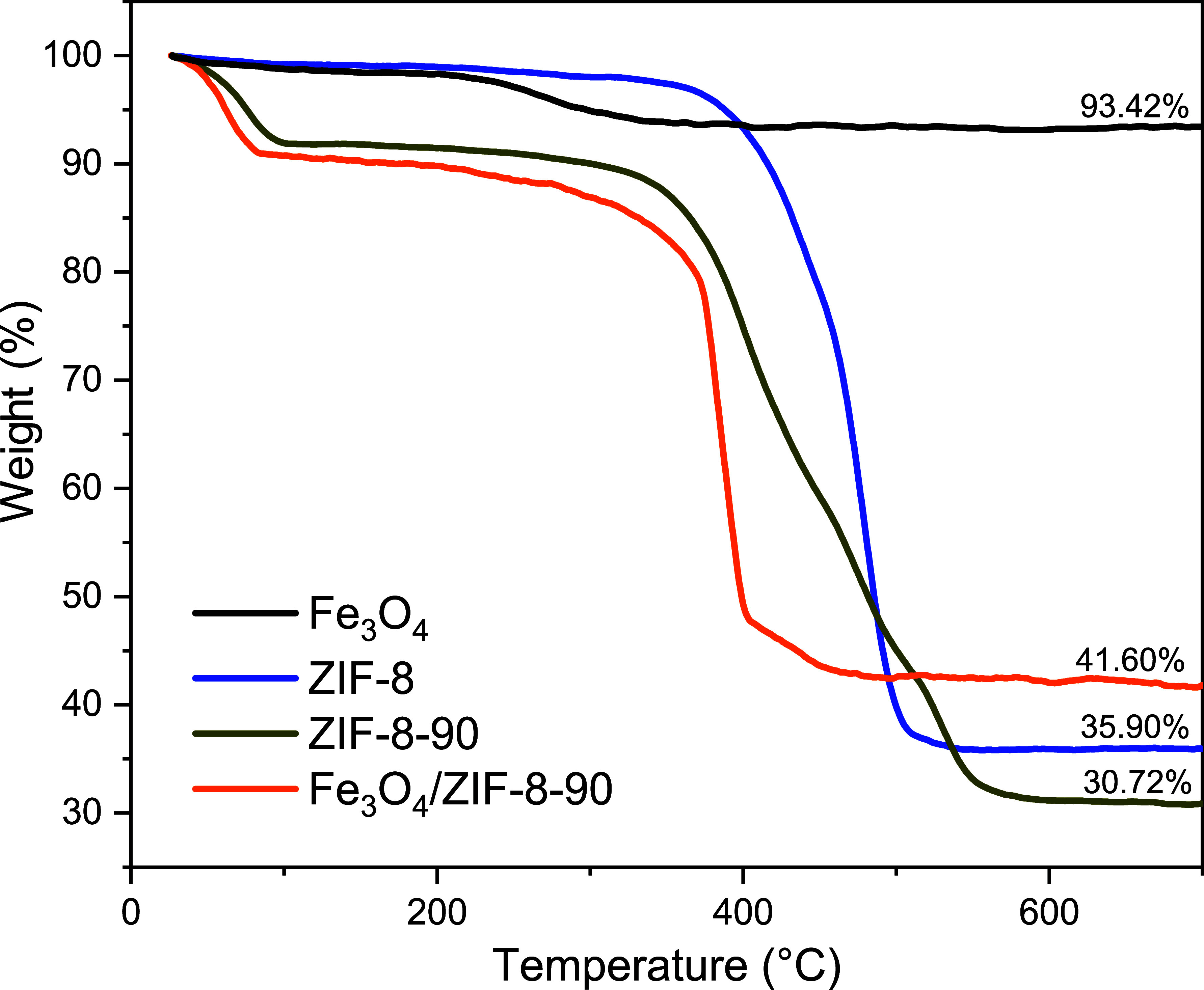
TGA curves for ZIF-8, ZIF-8-90, Fe_3_O_4_ and Fe_3_O_4_/ZIF-8-90.

For ZIF-8-90 and the Fe_3_O_4_/ZIF-8-90 composite, two main mass loss events were identified. The first event occurred at approximately 100 °C, with a mass loss of about 9%, attributed to the removal of guest molecules such as solvents or adsorbed water. The second event began at 300 °C and exhibited a two-step decomposition. The ICA ligand decomposed between 300 and 420 °C, resulting in a 23.45% mass loss. Subsequently, starting at 423 °C, the 2-MeIM ligand decomposed, causing an additional 35.43% mass loss. When compared to pure ZIF-8, it was observed that the presence of the ICA ligand in the ZIF-8-90 structure introduced new exothermic decomposition events (Figure S5b), consistent with the behavior described in the literature, as aldehydes oxidize more readily.^14,55^ This analysis suggests that the synthetic method employed resulted in a hybrid ZIF structure of approximately 60% 2-MeIM and 40% ICA (ZIF-8_60%_-90_40%_). For the Fe_3_O_4_/ZIF-8-90 composite (Figure 5), the TGA results exhibited similarities to the thermal profile of the pure ZIF-8-90 hybrid. However, the DTA analysis revealed a significant difference (Figure S5d). In the composite, thermal decomposition occurred as a single exothermic event, encompassing the degradation of both ligands, with a mass loss of 44.41%. This change in thermal behavior can be attributed to the interaction between the iron oxide nanoparticles and the ZIF-8-90 matrix, indicating the adhesion of the nanoparticles to the composite structure. This distinct thermal characteristic suggests a modification in the material’s thermal stability due to the incorporation of Fe_3_O_4_ nanoparticles. Considering that, on average, 30.72% of the remaining mass corresponds to ZnO, as previously observed in the pure hybrid ZIF, it is estimated that out of the 41.60%, 10.9% corresponds to the iron oxide nanoparticles.

### 2.2 Magnetic Properties

Figure 6 presents the magnetization curves of the NPs and the nanocomposites. For the SPIONs, the obtained specific saturation magnetization (Ms) value was 54.1 emu/g, that shows a superparamagnetic-like behavior under quasi-static conditions.^68^ The value is on the same order of other reports from the literature.^57,58^ The NPs showed a small coercivity value (Hc), 15 Oe, suggesting that a small number of larger NPs of the sample are at the blocked regime at room temperature.

**Figure 6:**
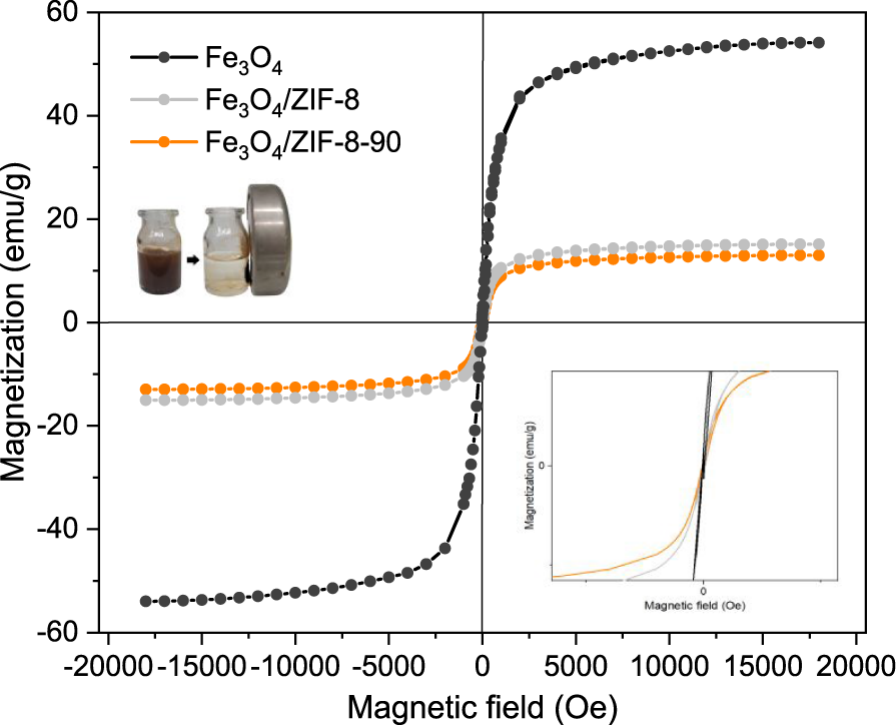
Magnetization curves of Fe_3_O_4_ NPs, for Fe_3_O_4_/ZIF-8 and Fe_3_O_4_/ZIF-8-90.

Upon incorporating Fe_3_O_4_ NPs into the ZIF-8 and, subsequently, ZIF-8-90 matrices, a reduction in both specific saturation magnetization (Ms) was observed. For Fe_3_O_4_/ZIF-8 Ms was 15.1 emu/g and Hc 26.27 Oe, while for Fe_3_O_4_/ZIF-8-90 Ms decreased to 13 emu/g and Hc varied to 28.91 Oe. The reduction in Ms is expected due to encapsulation of magnetite NPs in the ZIF nanostructure, and it only reflects the magnetic particle volume fraction of the nanocomposite. Note that in the experiment the magnetic moment value of the nanocomposite obtained by VSM is divided by the amount of composite mass, establishing the Ms value. There is no change in the NPs magnetic properties. Indeed, using the analysis of TGA that indicated that the composite has 10% of magnetic material, one can estimate the Fe_3_O_4_/ZIF-8 and Fe_3_O_4_/ZIF-8-90 composite density as 1.37 g/cm^3^ and 1.44 g/cm^3^, respectively. From this, it is possible to calculate the magnetic particle volume fraction of 7.31% for Fe_3_O_4_/ZIF-8 and 6.60% for Fe_3_O_4_/ZIF-8-90 incorporated in the nanocomposites.

Superparamagnetic nanoparticles (SPM) do not generate heat on their own, since heat generation is proportional to the hysteresis area.^33^ However, their behavior in the SPM regime is influenced by size, temperature, particle interaction, and the frequency of the applied AC magnetic field. According to Zufelato et al., at specific frequencies, a transition to the blocked regime may occur, enabling particle heating through dynamic hysteresis, which might be governed by mechanisms of collective magnetic relaxation.^25,28^ Thus, the hyperthermia test was also performed to evaluate the therapeutic potential of the Fe_3_O_4_/ZIF-8-90 nanocomposite.

### 2.3 Magnetic Hyperthermia Study

The magnetic hyperthermia analyses were performed under a field of 6.37 kAm^–1^ and a frequency of 323 kHz, with all experiments conducted at a concentration of 15 mg/mL (Figure 7).

**Figure 7:**
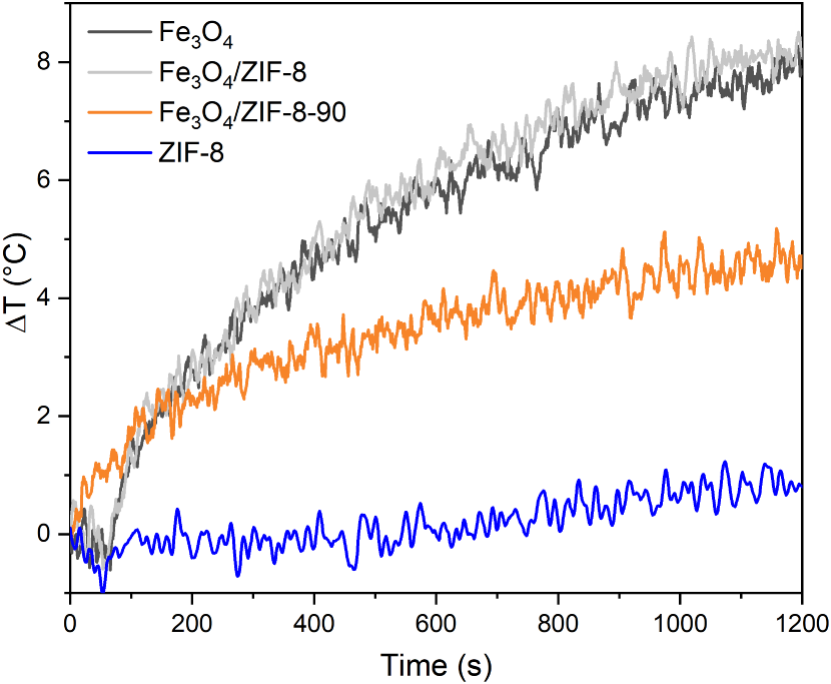
Magnetic hyperthermia curves over time for Fe_3_O_4_, Fe_3_O_4_/ZIF-8 and Fe_3_O_4_/ZIF-8-90, in front of a field of 80 Oe and frequency of 323 kHz.

Fe_3_O_4_ nanoparticles exhibited a temperature variation (Δ*T*) of 8.69 °C, while the Fe_3_O_4_/ZIF-8 composite showed slightly superior performance, with a Δ*T* of 9.08 °C. This increase can be attributed to the more efficient dispersion of Fe_3_O_4_ nanoparticles within the ZIF-8 matrix, which enhances the effective surface area and improves heat transfer. Additionally, the ZIF-8 matrix acts as an insulating medium, retaining the heat generated by the nanoparticles and thereby boosting overall thermal efficiency.^69^ In contrast, the Fe_3_O_4_/ZIF-8-90 composite exhibited a lower temperature variation (Δ*T* = 5.18 °C), which can be explained by the relatively lower concentration of Fe_3_O_4_ nanoparticles in the matrix, as evidenced by the decrease in particle volume fraction determined from VSM analysis.^70^

In terms of biological safety, the experimental conditions used in this study, with Hf = 2.06·10^9^ Am^–1^ s^–1^, are approximately two times below the safety threshold established by Dutz and Hergt (Hf ≤ 5·10^9^ Am^–1^ s^–1^).^26,71^ Nevertheless, even under these conditions, the results obtained provide a solid basis for estimating the magnetic hyperthermia potential of the studied materials, demonstrating their ability to generate sufficient heat to reach therapeutic temperatures (41–45 °C) for cancer treatment (or heat-induced drug release).

Table 1 compares the experimental magnetic hyperthermia setups described in the literature and the results obtained in this study. Although Fe_3_O_4_/MOF systems are not yet widely explored for magnetic hyperthermia applications, their potential is considerable.

**Table 1:** Experimental MH Configurations Reported in the Literature Compared with the Study Conducted in This Work

Magnetic Systems	Size (nm)	Field (kAm^–1^)	Frequency (kHz)	H × F (Am^–1^ s^–1^)	Concentration (mg/mL)	Δ*T*/Time	ref
Fe_3_O_4_@C	300	4.8	898	4.31 × 10^9^	2	43 °C/2 min	^74^
MNP@SiO_2_	116.8	15.92	307	4.9 × 10^9^	10	5 °C/20 min	^75^
Fe_3_O_4_@PDA@ZIF-90	200	14.32	409	5.9 × 10^9^	5	5.90 °C/20 min	^65^
Fe_3_O_4_/ZIF-8	76	6.37	323	2.1 × 10^9^	15	9.08 °C/20 min	Current study
Fe_3_O_4_/ZIF-8-90	97	6.37	323	2.1 × 10^9^	15	5.18 °C/20 min	Current study

Among the few examples identified, a notable system is Fe_3_O_4_@PDA@ZIF-90, evaluated by Chen et al., which achieved a Δ*T* of 5.9 °C using particle concentrations (5 mg/mL).^65^ However, the experimental conditions employed (Hxf = 5.9·10^9^ Am^–1^ s^–1^) exceeded the recommended biological safety limit, thereby constraining its clinical application.^72^ Additionally, the study by Udesh Dhawan et al. illustrates the combination of metallic nanoparticles (FeAu) encapsulated within multiple layers of MIL-100 (Fe) MOF.^73^ Although promising, this system was assessed under high-frequency induction waves (700–1000 kHz). In contrast, the materials developed in this study operated under biologically safe conditions, demonstrating thermal efficiency compatible with therapeutic requirements.

These results highlight the distinct advantages of the nanocomposites developed in this study. By incorporating a magnetic nanoparticle into a hybrid MOF (Fe_3_O_4_/ZIF-8-90), it was possible to efficiently explore an application that has been scarcely addressed in the magnetic hyperthermia literature. The ability of the composites to operate well below the biological safety limits while generating sufficient heat for therapeutic applications positions this study as a significant advancement in the use of MOFs for oncological treatments.

#### 2.3.1 Relaxometry

The ZIFs loaded with SPIONs exhibited increased sensitivity to magnetic resonance imaging, with relaxivity values of *r*_2_ of 161.21 mM^–1^ s^–1^ for Fe_3_O_4_/ZIF-8 and 180.15 mM^–1^ s^–1^ for Fe_3_O_4_/ZIF-8-90, values considerably higher than those of pure Fe_3_O_4_ nanoparticles (93.76 mM^–1^ s^–1^) (Figure 8a). The observed increase in *r*_2_ for Fe_3_O_4_/ZIF-8 and Fe_3_O_4_/ZIF-8-90 compared to pure Fe_3_O_4_ nanoparticles can be attributed to two main factors: (i) the aggregation effects on the surface of the ZIFs and (ii) the confinement-induced changes in water diffusion within the porous structures.^76^

**Figure 8:**
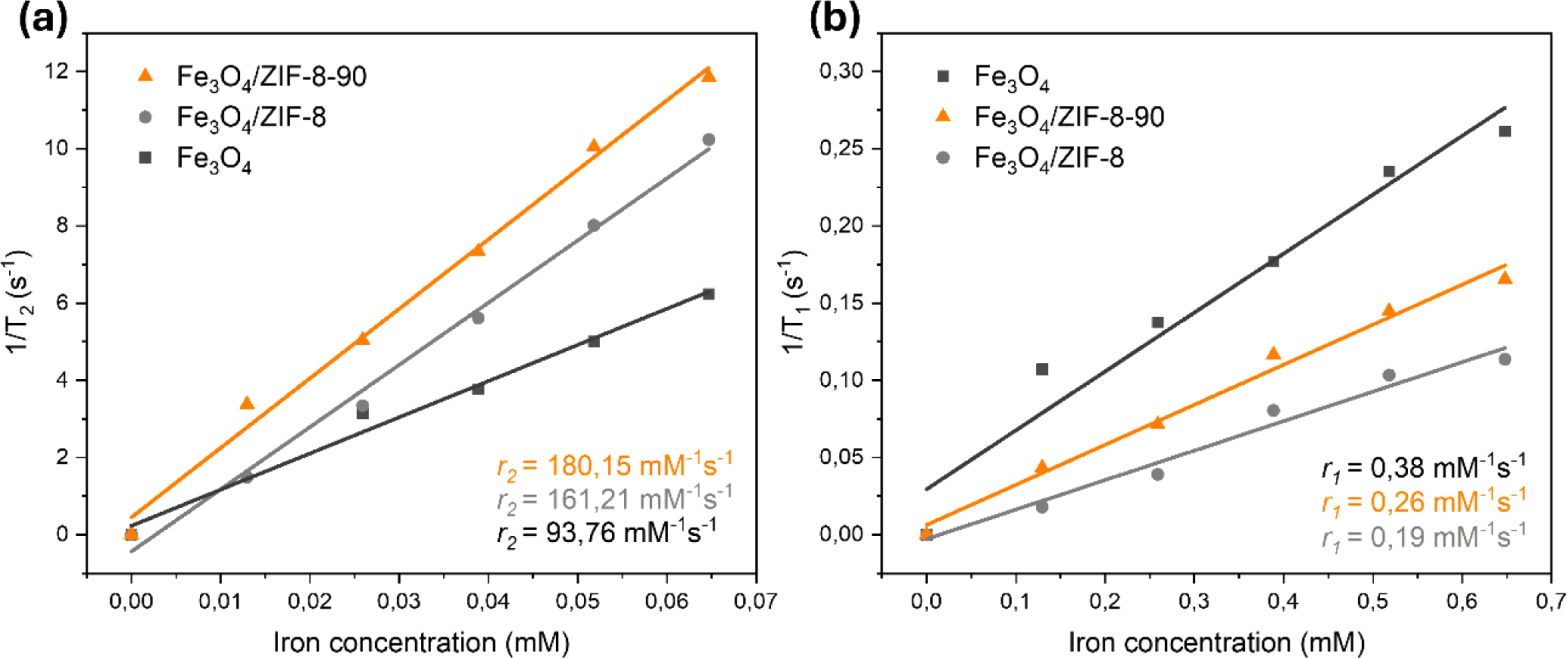
Variation of the transverse relaxation rate (a) (1/T_2_) and the longitudinal relaxation rate (b) (1/T_1_) as a function of Fe concentration. The measurements were carried out at 1.4 T, 60 MHz and 37 °C.

First, considering that superparamagnetic nanoparticles fall into the Motional Averaging Regime (MAR), where relaxivity is influenced by particle size and water mobility, the aggregation of Fe_3_O_4_ nanoparticles on the surface of ZIF-8 and ZIF-8-90 can lead to an increase in *r*_2_ due to the formation of larger hydrodynamic clusters.^77^ Studies have shown that as nanoparticle clusters’ hydrodynamic diameter (*Dh*) increases, the relaxivity *r*_2_ also increases until it reaches a critical size limit, beyond which the effect saturates.^78^

Subsequently, the porous nature of ZIF-8 and ZIF-8-90 introduces the diffusion of water molecules in confined environments. According to the outer-sphere relaxation theory, the diffusion coefficient of water (*Dw*) plays a crucial role in determining the *r*_2_ relaxivity.^79^ In porous materials, water molecules experience restricted mobility due to spatial confinement, leading to prolonged interactions with magnetic nanoparticles and an increase in *r*_2_. This effect was observed in hierarchical structures where MNPs were incorporated into mesoporous silicon and MOF matrices, significantly increasing relaxivity.^80–82^

Given that ZIF-8 and ZIF-8-90 exhibit well-defined porosity, the diffusion of water molecules within these structures likely follows similar confinement effects, further contributing to the observed increase in *r*_2_. Therefore, the combination of nanoparticle aggregation on the ZIF surface and altered water diffusion dynamics within the porous structure provides a plausible explanation for the enhanced transverse relaxivity of the Fe_3_O_4_/ZIF-8 and Fe_3_O_4_/ZIF-8-90 nanocomposites.

Additionally, as expected, the *r*_2_ value is significantly higher than *r*_1_ for all analyzed materials due to iron-based materials, such as SPIONs, being better T_2_ contrast agents.^83^ This data aligns with other works that use magnetic nanoparticles loaded in metal–organic frameworks, among other platforms (Table 2). The system cited by Mishra, S et al., for example, composed of mesoporous silica nanoparticles with iron oxide NPs, reaches *r*_2_ of 145.2 mM^–1^ s^–1^.^84^ In Liang, M et al., with a composite ZIF-8, Fe_3_O_4_@ZIF-8-Zn–Mn, a *r*_2_ of 21.28 mM^–1^ s^–1^ is achieved.^85^

**Table 2:** Values of Longitudinal (*r*_1_) and Transverse (*r*_2_) Relaxivities for Different Systems Compared with the Present Study

	Relaxivity (mM^–1^ s^–1^)			
Magnetic Systems	*r*_1_	*r*_2_	*B*_0_ (T)	ref
FA-FE-SBA15QN	-	145.2	3.0	^84^
Fe_3_O_4_@ZIF-8-Zn–Mn	0.82	21.28	0.5	^85^
USPIONs	20.5	157	1.4	^23^
Ferucarbotran	-	151	1.5	^88^
Combidex	-	65	1.5	^89^
Ferumoxytol	8.2	74.9	3.0	^86^
Feraheme	10.0	62.3	3.0	^86^
Fe_3_O_4_	0.38	46.18	1.4^a^	Current study
Fe_3_O_4_/ZIF-8	0.26	161.21	1.4^a^	Current study
Fe_3_O_4_/ZIF-8-90	0.19	180.15	1.4^a^	Current study

a All relaxivities were obtained at 37 °C.

Additionally, Fe_3_O_4_/ZIF-8 and Fe_3_O_4_/ZIF-8-90 synthesized in this work demonstrated higher *r*_2_ values than some commercial iron-based contrast agents. For instance, Ferucarbotran (Resovist) exhibits a relaxivity *r*_2_ of approximately 151 mM^–1^ s^–1^, while Combidex (*r*_2_ = 65 mM^–1^ s^–1^), Ferumoxytol (*r*_2_ = 74.9 mM^–1^ s^–1^), and Feraheme (*r*_2_ = 62.3 mM^–1^ s^–1^).^86–89^ This indicates that the developed nanocarrier is competitive and has great potential for use as a negative contrast agent in magnetic resonance imaging.

### 2.4 Adsorption and Release of 5-FU

The adsorption of 20 mg of the Fe_3_O_4_/ZIF-8-90 system showed a loading capacity of approximately 13% of the 30 mg of 5-FU, resulting in a 5-FU loading of 0.21 mg per milligram of material. Considering the results obtained by Kharen and Chandra, and by Li et al. in their studies on 5-FU cytotoxicity, 1 mg of the Fe_3_O_4_/ZIF-8-90/5-FU nanocomposite developed in this work would be sufficient to effectively induce tumor cell death in breast and lung cancer.^90–92^

The structural confirmation of the drug presence in the structure was performed by FTIR of the nanocomposite after adsorption and of the pure drug (Figure S6), where the presence of 5-FU in the system was observed through the presence of bands at 1243 cm^–1^ corresponding to in-plane C–N vibration and C–F stretching, and the band at 1647 cm^–1^ attributed to C=O bond vibrations.^93,94^

For the release, the analyses were carried out until a plateau was reached, as can be observed in Figure 9. In 1 h, approximately 20% of the drug load was released, and in the first 12 h, almost 90% of the release was achieved. The release was considered rapid due to the stimulation of drug diffusion into the medium by removing large aliquots. In 2 days, the release was practically complete (97%). It was noted that the release is gradual, which is a positive aspect of controlled treatment.

**Figure 9:**
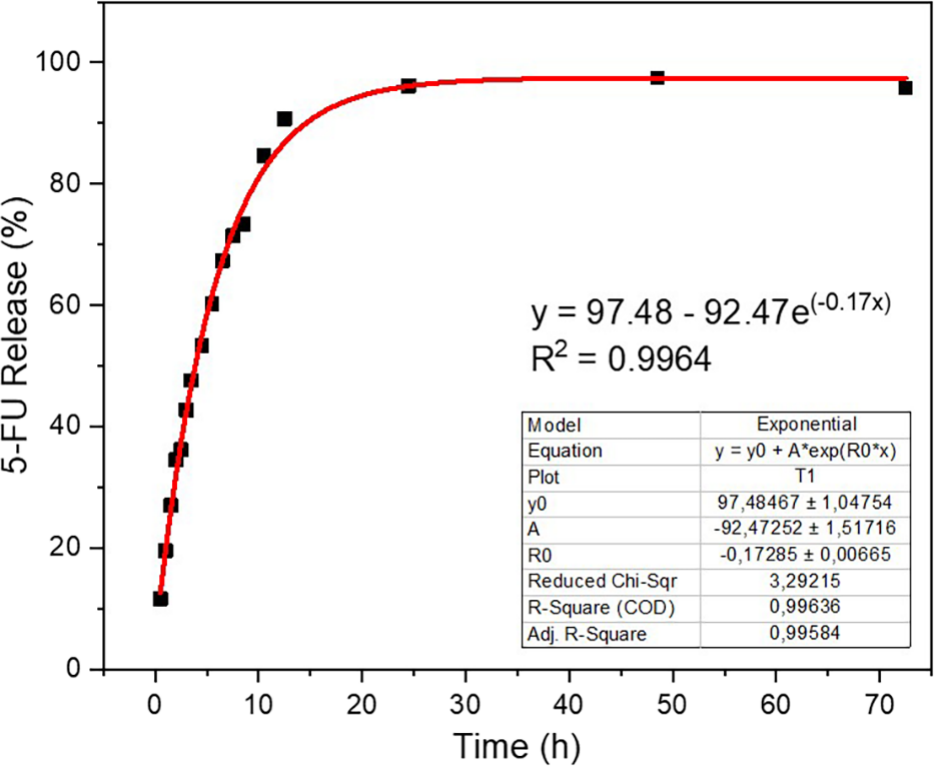
Release curve of 5-FU at pH 7.4.

### 2.5 Cytotoxicity Test

Tests conducted with the healthy Vero cell line demonstrated that both materials exhibited low toxicity, even at high concentrations, indicating their biocompatibility (Figure 10). In contrast, the materials showed a dose-dependent cytotoxic response in assays with tumor cells. For MDA-MB-231 cells, cell viability was reduced by approximately 40% at a 50 μg/mL concentration. Tumor cells H292, however, showed greater sensitivity to Fe_3_O_4_/ZIF-8-90, with a 39% reduction in cell viability at low concentrations of 0.7 μg/mL. This result surpasses some nanoparticle systems reported in the literature that generally require higher concentrations to achieve similar effects. For instance, the study by Santos et al. reported Fe_3_O_4_-based nanoparticles stabilized with silica, such as MNP@SiO_2_, which showed similar reductions in cell viability at higher concentrations, often well above 1 mg/mL for H292 lung tumor cells. These results reinforce the superior potential of Fe_3_O_4_/ZIF-8-90, which combines high cytotoxic efficiency with lower material concentrations, especially against the H292 cell line.

**Figure 10:**
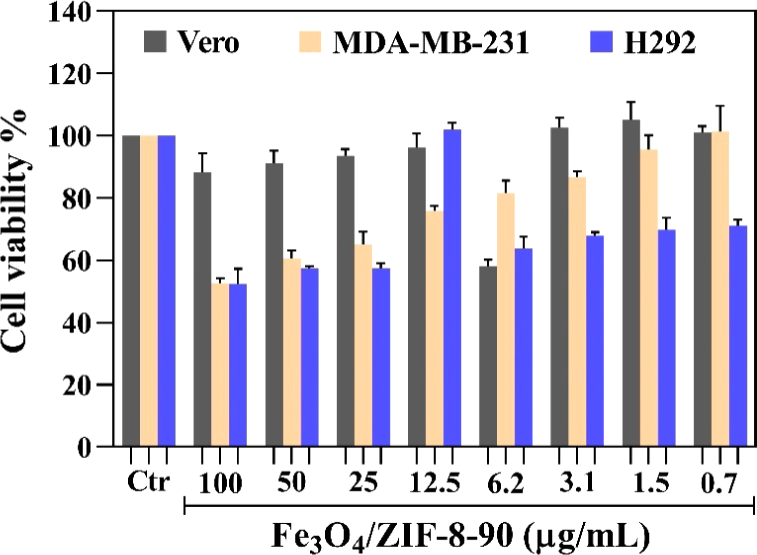
Viability of Vero cells, MDA-MB-231 and H292 tumor cells in the presence of Fe_3_O_4_/ZIF-8-90.

## 3 Conclusions

In this study, a multifunctional superparamagnetic nanocarrier, Fe_3_O_4_/ZIF-8-90, was developed based on a hybrid ZIF. The synthesis was optimized for reproducibility, resulting in a structurally stable material with a well-defined crystallographic pattern and an average particle size of 97.09 nm. The composite demonstrated a 5-FU loading capacity of 0.21 mg per milligram of material. In magnetic hyperthermia tests, Fe_3_O_4_/ZIF-8-90 exhibited a temperature elevation of 5.18 °C under alternating magnetic field conditions of 6.37 kAm^–1^ and a frequency of 323 kHz, remaining within the biological safety limits. Relaxometry tests indicated an increase in relaxivity (*r*_2_ = 180.15 mM^–1^ s^–1^), especially for T_2_-weighted images, reinforcing its potential as a contrast agent. Additionally, cell viability tests demonstrated the composite’s selectivity for MDA-MB-231 and H292 tumor cells, with a cytotoxic effect exclusive to these cells, while preserving the viability of healthy Vero cells.

Comparing the results to their equivalent Fe_3_O_4_/ZIF-8, Fe_3_O_4_/ZIF-8-90 exhibited superior performance in key aspects relevant to theranostic applications. A higher transverse relaxation rate suggests a more efficient material to act as a negative contrast agent, possibly due to the higher integration and dispersion of Fe_3_O_4_ nanocrystals within the ZIF-8-90 matrix. Additionally, although the thermal response observed in hyperthermia was moderate, the experiment was conducted under field conditions significantly below the clinical safety threshold, implying that heating efficiency may increase under more intense conditions. Importantly, using the carboxyl-functionalized ligand (ZIF-8-90) provides greater chemical versatility, facilitating future conjugation with targeting moieties and paving the way for developing more selective and personalized nanoplatforms. Therefore, Fe_3_O_4_/ZIF-8-90 emerges as a promising and adaptable theranostic candidate, integrating drug delivery, magnetic hyperthermia, and MRI contrast capabilities into a single nanosystem.

## 4 Materials and Methods

### 4.1 Chemicals and Reagents

2-Methylimidazole 99% (2-MeIM), imidazole-2-carboxyaldehyde 97% (ICA), 5-fluorouracil and polyvinylpyrrolidone (PVP, average molar weight: 10 000), all from Sigma-Aldrich. Zinc nitrate hexahydrate (Zn­(NO_3_)_2_·6H_2_O) P.A., oleic acid P.A., Iron­(III) chloride (FeCl_3_·6H_2_O), iron­(II) chloride (FeCl_2_·6H_2_O), ammonium hydroxide (NH_4_OH) (28–30% P.A.) all from Dinâmica. Absolute ethyl alcohol 99.8% P.A. and methyl alcohol 99.8% P.A., both from Química Moderna.

### 4.2 Instruments

Powder X-ray Diffractometry (XRD) analysis was performed using a Bruker eco D8 Advance device under a radiation source with a copper anode (CuKα (1.537 Å)). The morphology of the nanocomposite was characterized by a Tescan MIRA 3 scanning electron microscope (SEM) and a Tecnai G2 Spirit TWIN transmission electron microscope (TEM). Elemental mapping by the Energy Dispersive Detector (EDS) was performed by an Oxford Instruments Ultim Max 40 detector coupled to the SEM. For thermal analysis, a Shimadzu thermogravimetric analyzer, model TGA 60/60H, was used under a synthetic air atmosphere. Fourier transform infrared (FTIR) was performed using Shimadzu IRSpirit equipment with the ATR (attenuated total reflectance) accessory. The UV–vis absorption spectra were obtained on a Shimadzu UV-2600 spectrophotometer in the wavelength range of 200–800 nm. For magnetic characterization, a Vibrating Sample Magnetometer (VSM) ADE EV9 ADE-MAGNETICS model EV-9 operating in a magnetic field intensity range of −20 000 Oe to 20 000 Oe was used.

### 4.3 Synthesis of Iron Oxide Nanoparticles

SPIONs were synthesized via a modified coprecipitation method conducted under a nitrogen atmosphere to prevent iron ion oxidation.^23^ Briefly, a solution containing 3 g of iron­(III) chloride and 1.225 g of iron­(II) chloride was prepared in 12.5 mL of distilled water. After heating at 80 °C for 30 min, ammonium hydroxide (5.8 mL) was added dropwise as a precipitating agent, followed by oleic acid (0.532 mL). The reaction was allowed to proceed for an additional hour. After the formation of a black precipitate, indicating successful synthesis, the nanoparticles were washed three times successively with water and ethanol and then dried under vacuum at 30 °C.

### 4.4 Surface Modification of SPIONs with PVP

Based on the literature,^63^ 40 mg of PVP (Mw: 10 000) and 15 mg of presynthesized IO-NPs were added to distilled water in a single container and taken to an orbital shaker rotating at 150 rpm for 24 h. After this, the material was washed twice with water to remove excess PVP and dried under vacuum at room temperature.

### 4.5 Preparation of Fe_3_O_4_/ZIF-8

The following procedure was conducted: 405 mg of 2-MeIM and 368.5 mg of zinc nitrate hexahydrate were separately dissolved in 25 mL of methanol. 40 mg of SPIONs with PVP, redispersed in methanol, were added to the metal solution, followed by the previously prepared 2-MeIM solution. The system was agitated in an orbital shaker (150 rpm) for 5 min, resulting in a grayish coloration, and then left to rest for 24 h at ambient conditions. The final material was precipitated via centrifugation (5000 rpm), washed three times with methanol, and vacuum-dried at 30 °C.^5^

### 4.6 Preparation of Fe_3_O_4_/ZIF-8-90 Nanocomposite

Starting from the SALE (Solvent Assisted Linker Exchange) route,^95^ a 1:3 ratio (Fe_3_O_4_/ZIF-8:ICA linker) was used for the formation of the hybrid MOF ZIF-8-90. The Fe_3_O_4_/ZIF-8 system was redispersed in methanol using ultrasound, and the ICA, previously solubilized in methanol, was added dropwise to the dispersion. After the addition, the mixture was transferred to a Teflon reactor and placed in an oven at 60 °C for 3 days. Finally, the material was washed three times with methanol and dried in a vacuum oven at 30 °C.

### 4.7 Magnetic Hyperthermia Study

To evaluate the heating efficiency of the materials through magnetic hyperthermia, a MagneTherm 1.5 AC device from nanoTherics equipped with a LUXTRON 3300m fiber optic temperature probe from LumaSense Technologies was used. The magnetic heating variation over time was measured in suspensions with a concentration of 15 mg/mL for 20 min, starting at 25 °C. Measurements were performed in an AMF with a fixed frequency of 323 kHz under a magnetic field of 6.37 kAm^–1^ (80 Oe).

### 4.8 Relaxivity Measurements

Relaxometric measurements to determine the longitudinal and transverse relaxation times (T1 and T2) were conducted using aqueous solutions with five distinct concentrations of the nanomaterials. These measurements were carried out with a Bruker relaxometer operating at 60 MHz, using the Minispec mq60 model with a magnetic field of 1.41 T at 37 °C.

The relaxation rates were calculated as *R*_1_ = 1/*T*_1_ *R*_1_ and *R*_2_ = 1/*T*_2_ *R*_2_. The relaxivity values (*r*_1_ and *r*_2_) were determined from the linear fit of *R*_1_ and *R*_2_ as a function of the iron concentration [Fe] in the sample, following the equation:

1 $$R_{1}=R_{1,0}+r_{1}\left[\mathrm{Fe}\right]$$

2 $$R_{2}=R_{2,0}+r_{2}\left[\mathrm{Fe}\right]$$

where *R*_1,0_ and *R*_2,0_ are the intrinsic relaxation rates of the medium in the absence of the nanomaterial, and *r*_1_ and *r*_2_ are the relaxivities, which describe the efficiency of the nanomaterial in altering the relaxation properties of the medium.

### 4.9 Loading and Release of 5-FU from Fe_3_O_4_/ZIF-8-90 Nanocomposite

The 5-FU was incorporated into the system at a 3:2 molar ratio of 5-FU to Fe_3_O_4_/ZIF-8-90, as previously described.^96^ To achieve this, 20 mg of the system was added to a 5 mL methanolic solution containing the drug. The mixture was subjected to orbital shaking for 48 h to facilitate adsorption. Subsequently, the sample was centrifuged for 10 min to isolate the loaded system. The loading capacity was determined using the following equation:

3 $$\mathrm{Loading content}\left(\%\right)=\frac{\left(m_{1}-m_{2}\right)100\%}{m_{1}}$$

Where *m*_1_ (mg) is the mass of the drug before adsorption, and *m*_2_ (mg) is the mass of the drug remaining in solution after 48 h of adsorption. The release was carried out in phosphate-buffered saline (PBS) solution at pH 7.4, at 37 °C, and with a rotation speed of 50 rpm in a dissolution apparatus. Several samples were collected at different time points, and their concentrations were determined by UV–vis spectroscopy (λmax = 266 nm for 5-FU). Each experiment was performed in triplicate, and the reported values are the mean values.

### 4.10 MTT Assay

To determine the cytotoxicity of ZIF-8-90 and Fe_3_O_4_/ZIF-8-90 samples, healthy epithelial cells (VERO) and cancer cell lines of breast cancer (MDA-MB-231) and lung cancer (H292) were used. These cells were cultured in RPMI-1640 medium supplemented with 10% fetal bovine serum (FBS) and 1% penicillin/streptomycin, maintained in an incubator at 37 °C and 5% CO_2_. For the assays, cell suspensions were seeded in 96-well plates (1 × 10^4^ cells/well) and treated with different concentrations of the samples (100 to 0.7 μg/mL) for 24 h. After incubation with the treatment, 10 μL of MTT (3-(4,5-dimethylthiazol-2-yl)-2,5-diphenyltetrazolium bromide) diluted in PBS (5 mg/mL) was added to each well and incubated for 3 h in an incubator at 37 °C and 5% CO_2_. Subsequently, 100 μL of the solubilization solution was added to dissolve the formazan crystals. Then, cell viability was measured optically (at 570 nm). All assays were performed in triplicate, the obtained data were analyzed using ANOVA for comparison between groups, and differences were evaluated by one-way posthoc test (*p* < 0.05).

## Supplementary Material



## Notes

Crystallographic details for the synthesized materials; data from semiquantitative analysis of mass percentages for carbon, zinc, nitrogen, oxygen, and iron; estimated values for 5-FU adsorption in systems with and without SPIONs; graphs generated after Rietveld Refinement of each material through GSAS-II; SEM images; TEM image for Fe_3_O_4_ NPs; sum-map spectra for EDS associated with layered image; TGA and DTA curves; FTIR spectrum of the nanocomposites after the adsorption of 5-FU and the pure drug; and viability of Vero cells, and MDA-MB-231 and H292 tumor cells in the presence of ZIF-8-90 (PDF)
