# Supplementary material for: Fe 3 O 4 /ZIF-8-90 Nanocomposite as a Strategy for Oncological Treatment
Source: ACS Omega. 2025 Jul 3;10(27):29463–75. doi: 10.1021/acsomega.5c02819 (PMC12268462; doi:10.1021/acsomega.5c02819)
Supplement: Supplementary file 1 [file ao5c02819_si_001.pdf]

Supporting Information

Fe_3O_4/ZIF-8-90 Nanocomposite as a strategy for oncological treatment

Julia Fernanda da Costa Araujo[a], Giovanna Nogueira da Silva Avelino Oliveira Rocha[a], José Yago Rodrigues Silva[a*], João Victor Ribeiro Rocha[b], Andris Figueiroa Bakuzis[b], Severino Alves Junior[a*]

[a]Department of Fundamental Chemistry, Federal University of Pernambuco, Recife, PE, Brazil

[c]Physics Institute, Federal University of Goias, Goiania, Brazil

Supporting Information

Fe_3O_4/ZIF-8-90 Nanocomposite as a strategy for oncological treatment

Julia Fernanda da Costa Araujo[a], Giovanna Nogueira da Silva Avelino Oliveira Rocha[a], José Yago Rodrigues Silva[a*], João Victor Ribeiro Rocha[b], Andris Figueiroa Bakuzis[b], Severino Alves Junior[a]*

[a]Department of Fundamental Chemistry, Federal University of Pernambuco, Recife, PE, Brazil
[c]Physics Institute, Federal University of Goias, Goiania, Brazil

* Corresponding author: Severino Alves Junior and José Yago Rodrigues Silva

Table S1. Crystallographic details for Fe_3O_4, ZIF-8, ZIF-8-90 and Fe_3O_4/ZIF-8-90

Parameters	Materials			
	**Fe_3O_4**	**ZIF-8**	**ZIF-8-90**	**Fe_3O_4/ZIF-8-90**
**Empirical formula**	Fe_3O_4	$Zn(C_4H_6N_2)_2$	$Zn(C_4H_6N_2)_{0.6}$ $(C_4H_4N_2O)_{0.4}$	$Fe_3O_4/Zn(C_4H_6N_2)_{0.6}$ $(C_4H_4N_2O)_{0.4}$
**Molar mass (g/mol)**	231.533	229.592	152.078	160.923
**Crystal system**	Face-centered cubic (FCC)	Body-centered cubic (BCC)	Body-centered cubic	Body-centered cubic
**Space group**	*F*d-3m	*I*-43m	*I*-43m	*F*d-3m / *I*-43m
a (Å)	8.37024	17.04305	17.06042	8.37344/17.04597
b (Å)	8.37024	17.04305	17.06042	8.37344/17.04597
c (Å)	8.37024	17.04305	17.06042	8.37344/17.04597
α (°)	90	90	90	90
β (°)	90	90	90	90
γ (°)	90	90	90	90
V ($Å^3$)	586.426	4950.421	4965.572	587.099 /4952.960
Z	8	12	19	
λ (Å)	1.540500 1.544300	1.540500 1.544300	1.540500 1.544300	1.540500 1.544300
d_{calc} (g/cm^3)	5.2450	0.9331	0.9748	5.2390 / 0.9545
**Crystallite size *(nm) (GSAS-II Fit)***	12.2			
**Crystallite size *(nm) (Williamson-Hall)***	13.59	59.44	68.07	87.88
h, k, l max	7, 5, 4	7, 5, 4	7, 5, 4	7, 5, 4
N_{ref}	21	36	36	36
θ_{min} ; θ_{max} (°)	5; 90	5; 40	5; 40	5; 40
N° of Phases	1	1	1	2
Phase fraction (%)	100	100	100	41.165 / 58.835
Weight fraction (%)	100	100	100	31.282 / 68.718
wR2	21.009	10.667	21.059	13.179
GOF	2.02	4.39	7.33	2.64

Table S2. Data from semi-quantitative analysis of mass percentages for carbon (C), zinc (Zn), nitrogen (N), oxygen (O) and iron (Fe) in the compounds Fe_3O_4, ZIF-8, ZIF-8-90, Fe_3O_4/ZIF-8 and Fe_3O_4/ZIF-8-90.

Sample	Weight (%)				
	**Carbon**	**Zinc**	**Nitrogen**	**Oxygen**	**Iron**
Fe_3O_4	10.6	-	-	33.3	56.1
ZIF-8	46.4	27.4	26.1	-	-
ZIF-8-90	47.8	27.9	20	4.4	-
Fe_3O_4/ZIF-8	46.7	17.4	25.7	6	4.3
Fe_3O_4/ZIF-8-90	32.4	44.44	3.2	2.7	17.2

Table S3. Estimated values for 5-FU adsorption in systems with and without SPIONs.

Material	Adsorption time (start/end)	Triplicate (ppm)				Total adsorbed (%)
		1	*2*	*3*	*Average*	
ZIF-8-90/5-FU	0 h	9.6822	10.0748	9.9199	9.8926	25.80
	48h	6.7300	8.1388	7.1523	7.3404	
Fe_3O_4/ZIF-8-90/5-FU	0 h	10.4617	10.1100	11.7032	10.7583	13.64
	48 h	9.0090	8.8142	10.0493	9.2908	

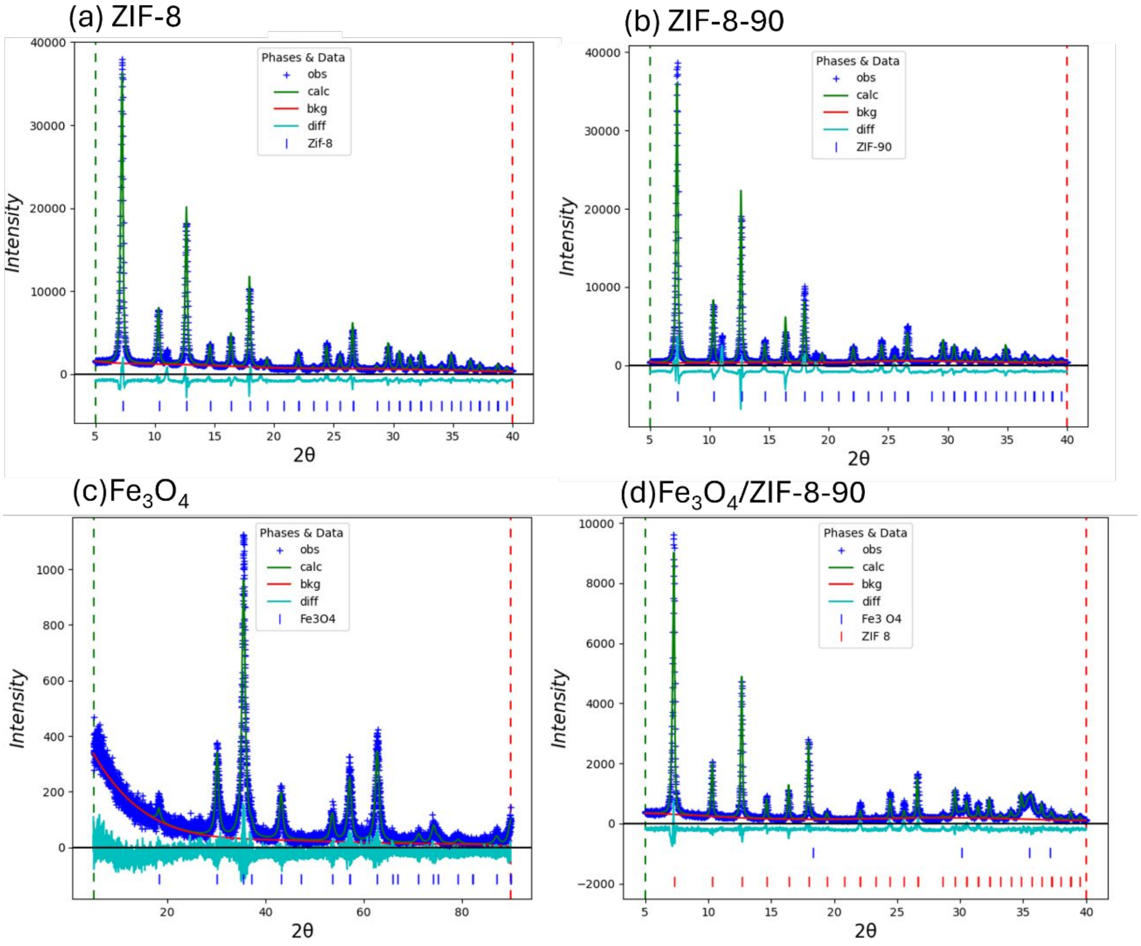

Figure S1. Graphs generated after Rietveld Refinement of each material through GSAS-II, a) ZIF-8, (b) ZIF-8-90, (c) Fe_3O_4 and (d) Fe3O4/ ZIF-8-90

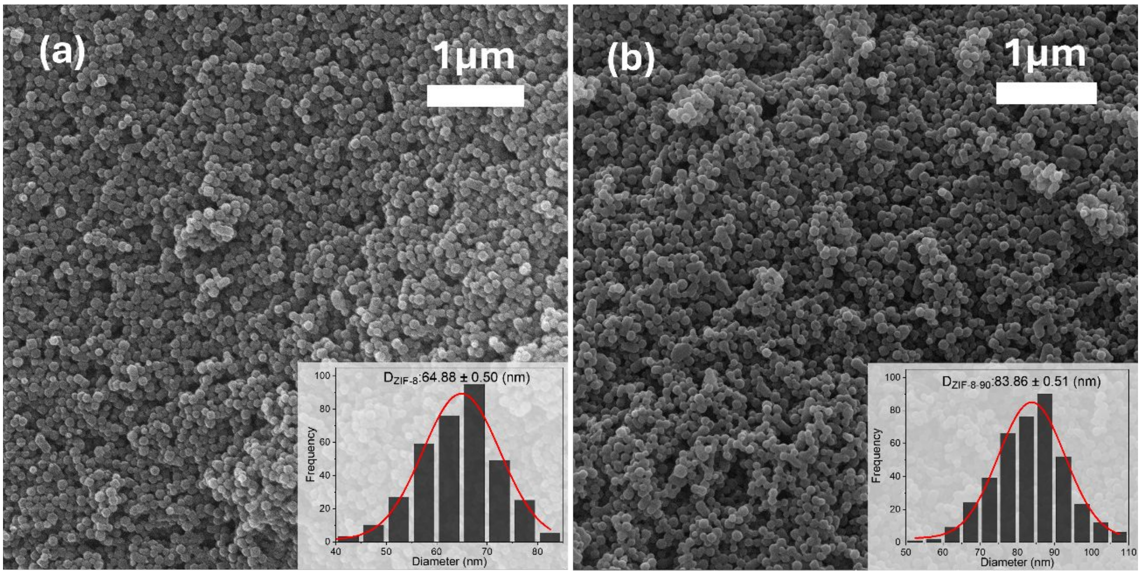

Figure S2. SEM images of (a) ZIF-8 (64.88 ± 0.50 nm) and (b) ZIF-8-90 (83.86 ± 0.51 nm).

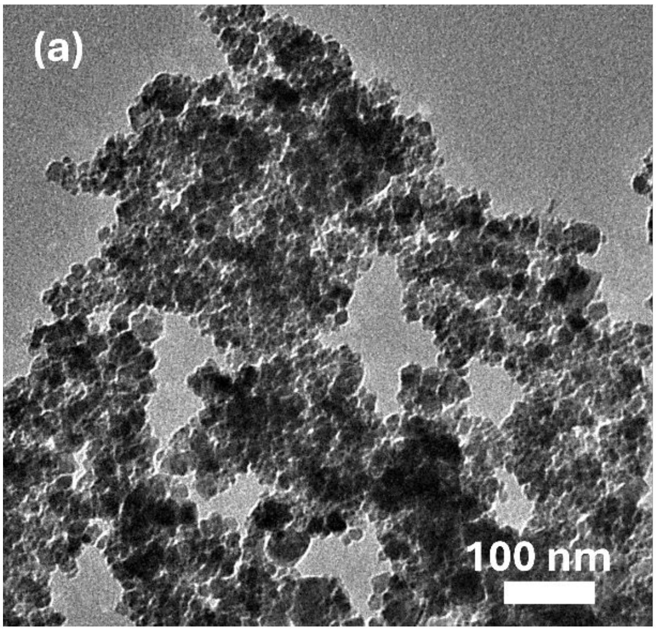

Figure S3. TEM image for (a) Fe_3O_4 NPs.

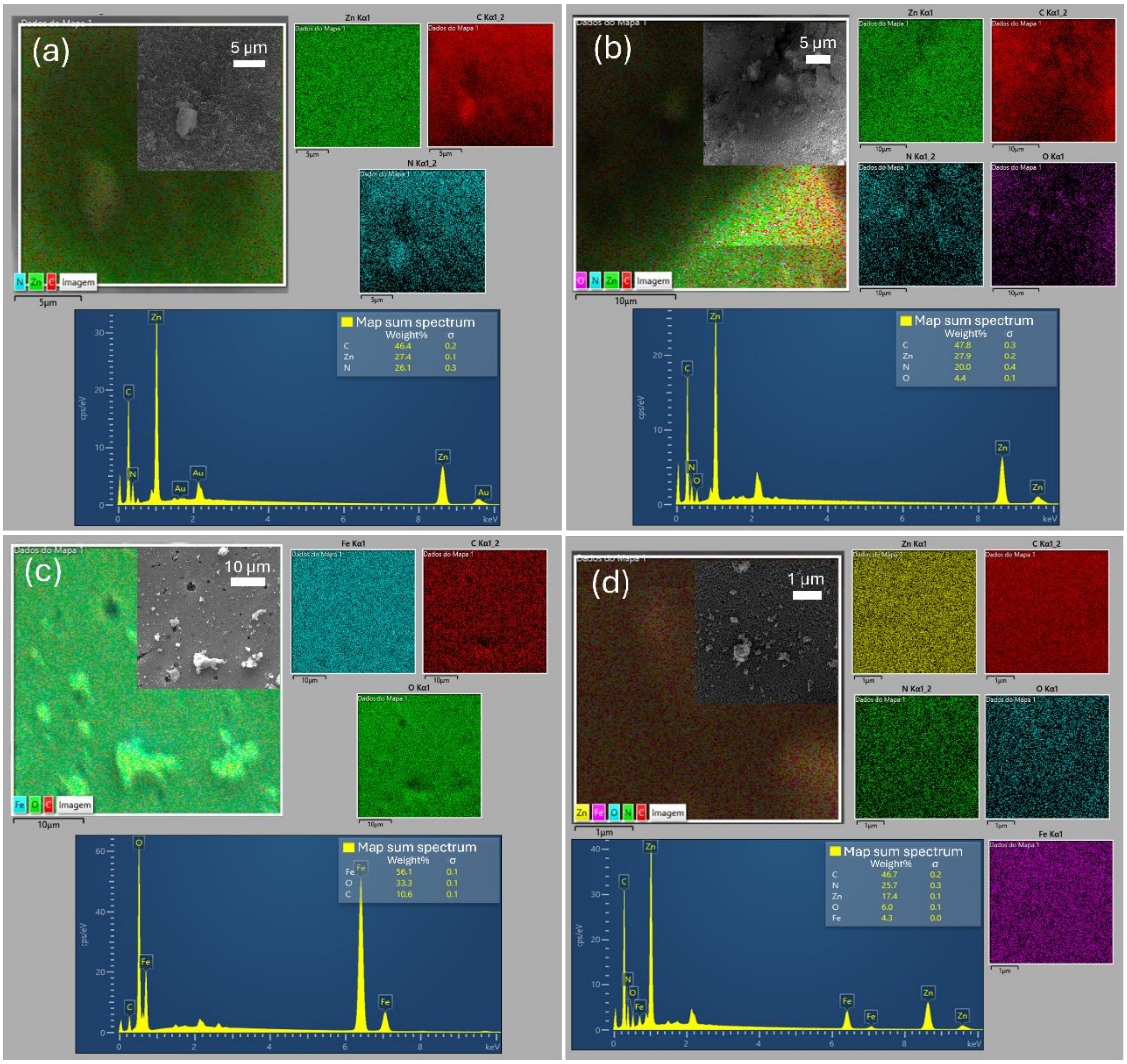

Figure S4. Sum-map spectra for EDS associated with layered image of (a) ZIF-8, (b) ZIF-8-90, (c) Fe_3O_4 and (d) Fe_3O_4/ZIF-8-90.

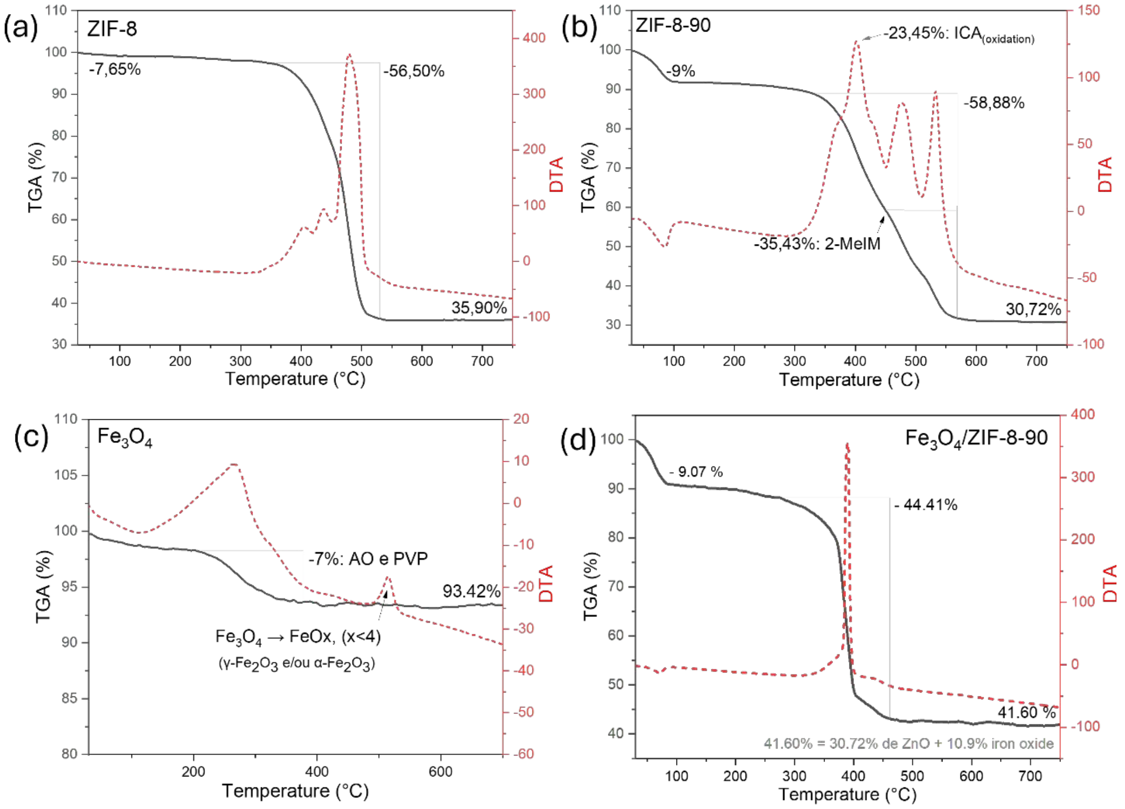

Figure S5. TGA and DTA curves for (a) ZIF-8, (b) ZIF-8-90, (c) Fe_3O_4, and (d) Fe_3O_4/ZIF-8-90 with the respective mass losses described.

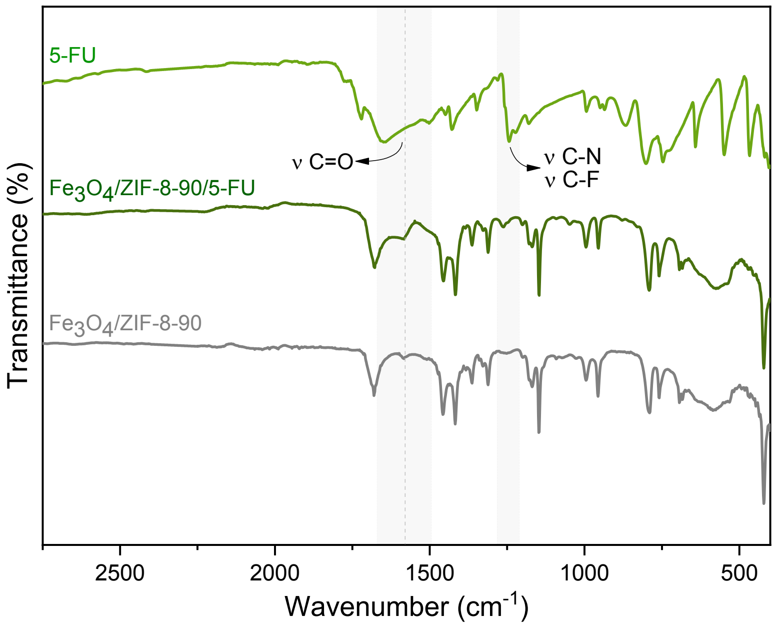

Figure S6. FTIR spectrum of the nanocomposites after the adsorption of 5-FU and the pure drug.

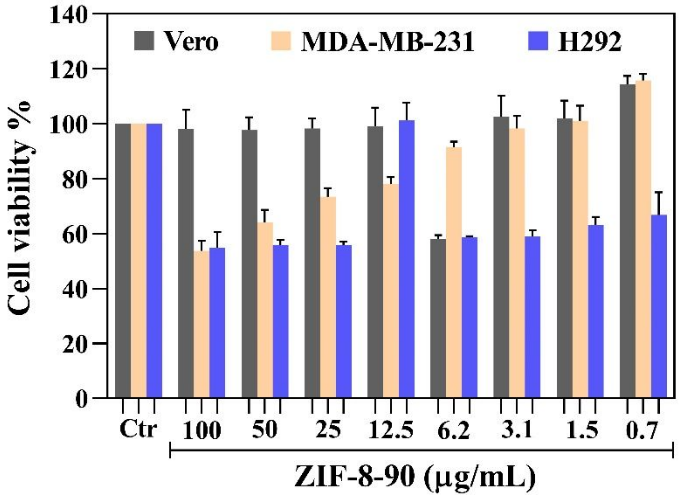

Figure S7. Viability of Vero cells, and MDA-MB-231 and H292 tumor cells in the presence of ZIF-8-90.
